# The use of image synthesis techniques in target and roi delineation in the upright position

**DOI:** 10.1002/acm2.14079

**Published:** 2023-06-22

**Authors:** Andries Niek Schreuder, Nikos Paragios, Michael Kissick, Michelle Lis, Tracy S. A. Underwood, Rockwell Mackie

**Affiliations:** ^1^ LEO Cancer care Middleton Wisconsin USA; ^2^ TheraPanacea Paris France; ^3^ Department of Medical Physics and Biomedical Engineering Leo Cancer Care London UK

**Keywords:** synthetic images, upright imaging, upright radiation therapy

## Abstract

The use of multi‐modality imaging technologies such as CT, MRI, and PET imaging is state of the art for radiation therapy treatment planning. Except for a limited number of low magnetic field MR scanners the majority of such imaging technologies can only image the patient in a recumbent position. Delivering radiation therapy treatments with the patient in an upright orientation has many benefits and several companies are now developing upright patient positioners combined with upright diagnostic helical CT scanners to facilitate upright radiation therapy treatments. Due to the directional changes in the gravitational forces on the patient's body, most structures and organs will change position and shape between the recumbent and upright positions. Detailed knowledge about such structures and organs are therefore often only available in the recumbent position. The problem statement is therefore well defined, that is, how do we know where such structures and organs, that is, the target or region at risk volumes, are in the upright position if those cannot be identified and or delineated accurately enough using the upright diagnostic quality CT images only? This paper outlines two methods based on synthetic CT or MR images to overcome this problem.

## 1 INTRODUCTION

**FIGURE 1:**
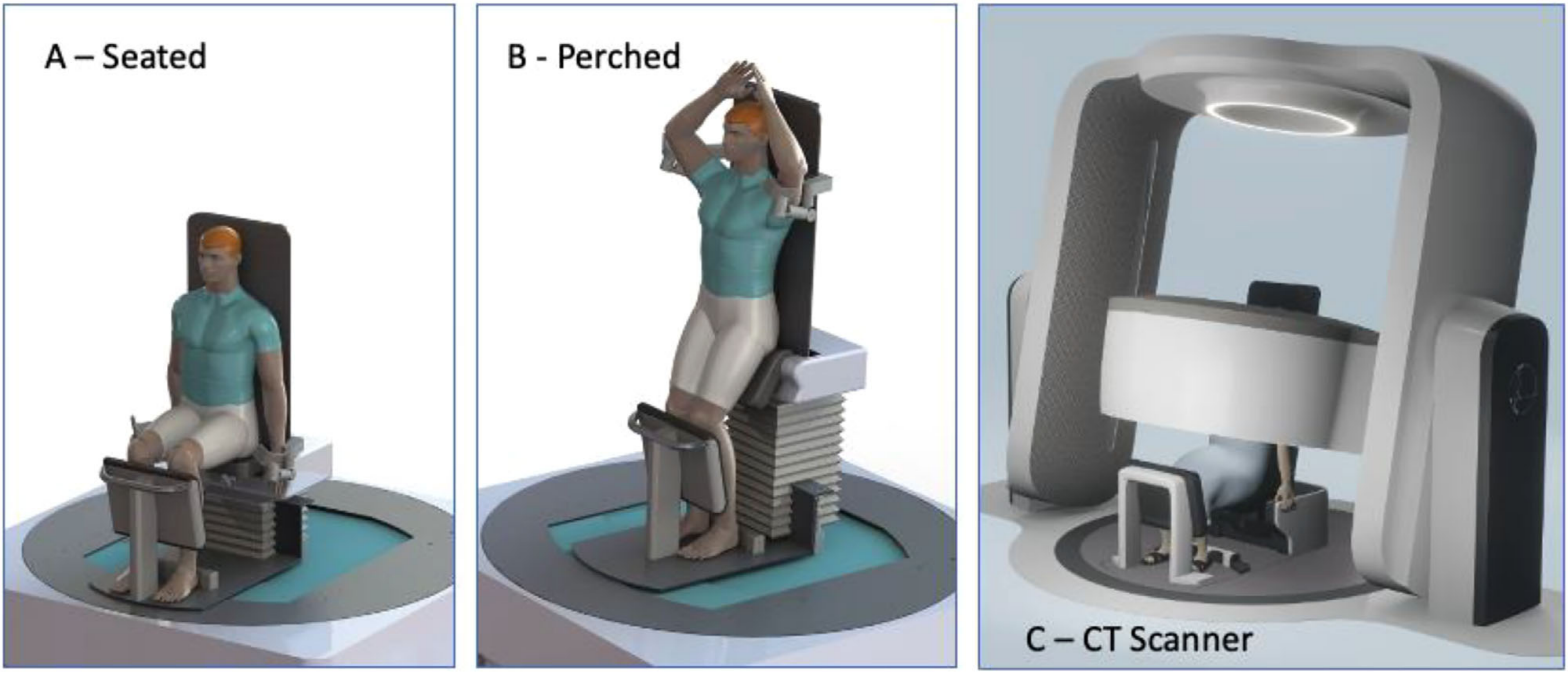
Schematic drawings of the Leo Cancer Care patient positioner and the upright CT scanner.

In the early days of radiation therapy, patients were treated mostly in and upright orientation (seated position).^1^ However, due to many late diagnoses of cancers and subsequent bad prognoses, many patients could not tolerate the upright orientation and which favoured procedures in the recumbent position. As a result of vastly improved diagnostic imaging technologies which enables early detection of cancers, cancer patients are now generally more ambulatory allowing for a resurgence of upright imaging and treatment procedures. Several benefits of upright imaging and radiotherapy treatments have been researched and documented in recent years.^2^, ^3^, ^4^ Reserachers at Keio University school of Medicine in Japan recently developed an upright CT scanner allowing for diagnostic CT scans with the patient in a standing position.^5^ They list many clinical benefits of imaging patients in the upright orientation. Leo Cancer Care brings some of these benefits to a clinical realization by developing technologies that will enable positioning, immobilization and imaging patients in an upright orientation. These systems can be integrated with multiple radiation therapy modalities such as x‐rays, electrons, neutrons, and accelerated particle beams to allow upright radiation therapy treatments hence standardizing patient positioning and imaging across these modalities. A sophisticated upright patient positioner has been developed and is now undergoing clinical testing at a customer site. Bosbouvier et al. concluded that the patient positioner can comfortably, precisely and reproducibly position the patient in a semi‐standing position for targets in the pelvic region.^6^ In addition to the patient positioner, a diagnostic quality, upright dual energy CT (DECT) scanner has also been developed to image the patient in the upright position. The upright DECT scanner comprises of a 32 slice axial CT scanner ring mounted on a gantry structure with support arms that can tilt about a horizontal axis while allowing the CT ring to be translated along the arms using precision slide rails. The CT scanner has a 63 cm diameter radiation Field of View (FOV) and an 85 cm bore diameter. The CT ring rotates at a maximum speed of 60 rpm and can travel over a distance of 1.75 m while scanning, that is, from the top position to the lowest position, which allows for scanning a very large volume. The x‐ray tube energy can be varied in 10 KVP steps between 80 and 140 KVP and with a maximum of 240 mA filament current. The maximum x‐ray tube power is 42 kW. The tilt angles of the CT support arms are limited to ± 15 degrees off vertical and are synchronized with the tilt of the backrest of the patient positioner to ensure that the CT scan plane is always perpendicular with the backrest and hence the long axis of the patient. Schematic drawings of the patient positioner and the accompanying CT scanner are shown in Figure 1.

In order to facilitate upright radiotherapy treatments for any targets outside the cranium, a CT scan of the patient must be acquired in the upright position. This is feasible since the movement and deformation of intracranial targets between the recumbent and upright orientations are fairly small^7^ and are typically accommodated by the uncertainty margins applied when treating intracranial targets in the upright position without upright imaging.^8^ Once a CT scan is acquired in the upright position using the upright CT scanner, the patient positioner can be used to position patients in the upright position in front of any radiation therapy beam—typically a fixed horizontal beam. The image guidance for positioning the patient can be done using an upright CT scanner, static planar x‐rays, or KV cone beam CT (CBCT) acquired through rotating the patient in a stationary KV x‐ray beam.

Using multi‐modality imaging technologies such as CT, MRI, and PET imaging is state of the art for radiation therapy treatment planning. Modern MRI scanners use magnetic fields strengths of more than 1.5T. High field strength MRI images are often preferred by diagnostic radiologists and radiation oncologists to determine the exact location and shape of the target to be treated and the critical structures that must be spared from radiation. Currently, all high‐field MRI imaging technologies can only image the patient in a recumbent position, that is, the supine, prone or decubitus positions. Today, less than 200 upright MRI scanners are in operation worldwide and none of them offer magnetic field strengths higher than 0.6 T.^9^

Due to the directional changes in the gravitational forces on the patient's body, most structures and organs will change position and shape between the recumbent and upright positions. The problem statement is therefore well defined, that is, how do we know where target or region at risk volumes are in the upright position if many cannot be identified and/or delineated accurately enough using diagnostic quality CT images only? This technical note proposes methods to mitigate this potential limitation.

## 2 IMAGE REGISTRATION

Image registration merges visual information from two or more imaging modalities.^10^, ^11^ Image registrations can be divided into two broad categories, that is, intra‐ and inter‐modality registrations. Intra‐modality registrations are image registrations for the same imaging modality, for example, CT to CT or MRI to MRI while inter‐modality registrations are between two different imaging modalities, for example, MRI to CT or PET to CT. Image registration methods for either intra‐ or inter‐modality registrations are normally categorized into rigid and deformable (non‐rigid/elastic) methods. Rigid registrations only allow for rotations and translations between the imaging modalities under consideration. Deformable image registrations relies on elastic mapping processes that are based on matching observed anatomical structures across imaging modalities under consideration.^12^ Image registration techniques and algorithms received a lot of attention from many scholars with the primary focus on adaptive radiation therapy.^13^, ^14^ Adaptive radiation therapy is required for three fundamental reasons, that is, (1) to accommodate changes in the patient geometry due to setup errors or setup difficulties including motion, (2) anatomical changes in the treatment region due to physiology, for example, heart beating or respiratory motion and or tumor growth or shrinkage, and (3) anatomical changes in the treatment region due to patient weight loss or gain. All these changes take place with the patient in the same orientation and relative position. Inter‐ and intra‐modality image registrations between images with the patient in totally different orientations such as recumbent to upright bring a new set of challenges.

**FIGURE 2:**
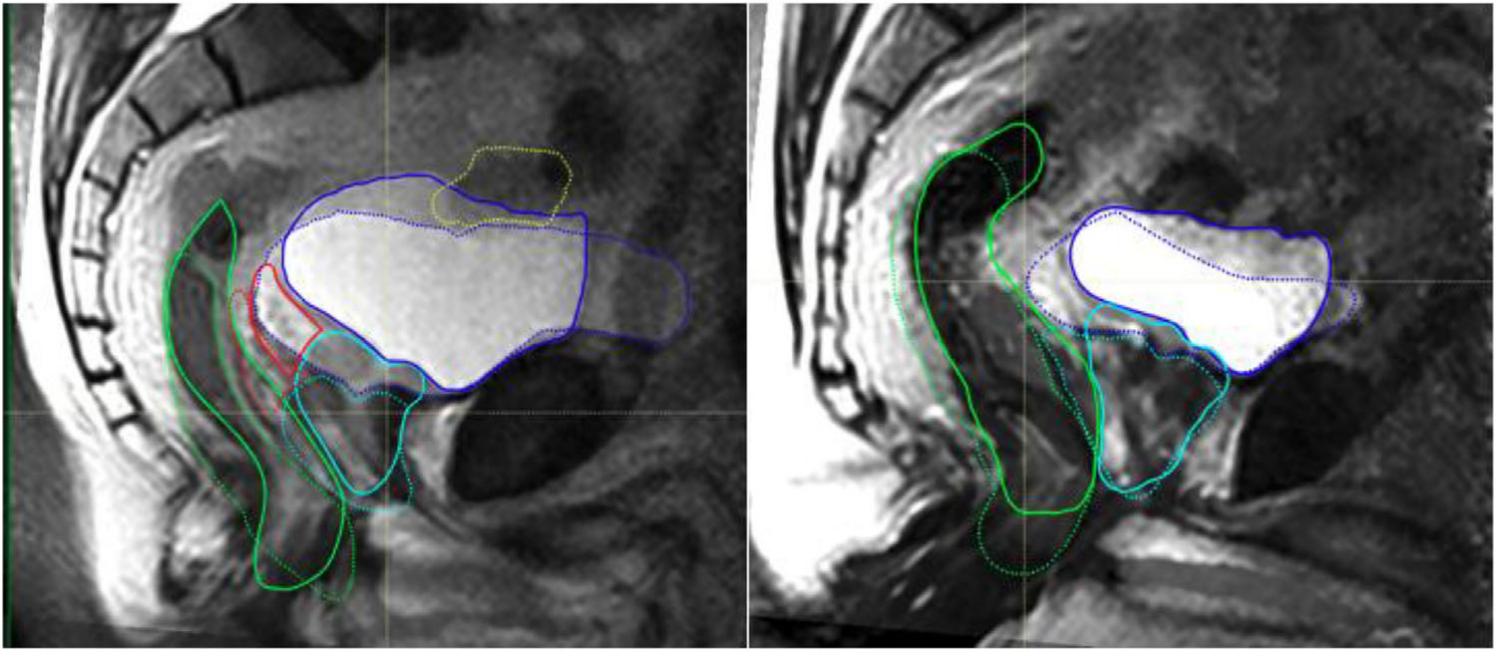
The midline sagittal MRI images for the supine (solid lines) upright (dotted lines) positions for two volunteers. The following organs are shown in both panels—rectum (green), prostate (light blue), bladder (blue) and small bowel (yellow—left panel only).

Significant anatomical changes have been observed for certain anatomical regions such as the kidneys,^15^ the liver,^16^ pelvic floor,^17^ and other thoracic organs.^18^, ^19^ A recent study comparing male pelvic organs between the supine and upright orientations revealed that there were large changes in the shape and position of the bladder while the shape of the prostate did not change significantly.^20^ This is illustrated in Figure 2 for two of the volunteers scanned in the study. When we investigated the use of normal deformable registration techniques, it was evident that the large deformation required to match the bladder adversely impact the shape of the prostate. One way to mitigate this is to crop the region of interest and only deform a smaller volume into the upright geometry.

Intra‐modality registrations are more tractable than inter‐modality registrations and due to the similarity of the observed signals it typically provide more accurate and trustworthy registrations. A simple explanation for this is that image registration algorithms involve three components: the deformation model, the similarity metric and the optimization strategy. The similarity metric, or the way the algorithms compare the signals from the images under consideration, changes completely for inter‐modality registrations. For intra‐modality registrations, the algorithms can use robust comparisons on a voxel‐by‐voxel basis that aggregate the local information. For inter‐modality registrations, the algorithms must rely on global statistical consistency across distributions, and the aim is to find a transformation that establishes distribution relevancies, which are much harder to quantify locally^10^, ^11^ due to the limited number of samples

## 3 IMAGE SYNTHESIS

Recent progress in the field of artificial intelligence (AI) and specifically in the domain of image synthesis using deep generative neural networks it is now possible to calculate bijective transformations between pairs of images such as synthetic CT from MRI and vice versa.^21^, ^22^, ^23^ Such a transformation can generate a synthetic image for the missing modality, that is, using the image data set acquired in the desired orientation to calculate the synthetic image set. This allows for improved intra‐modality registrations, that is, CT to CT or MRI to MRI where in the past one would have to rely on weaker inter‐modality, that is, CT to MRI or MRI to CT registrations.

**FIGURE 3:**
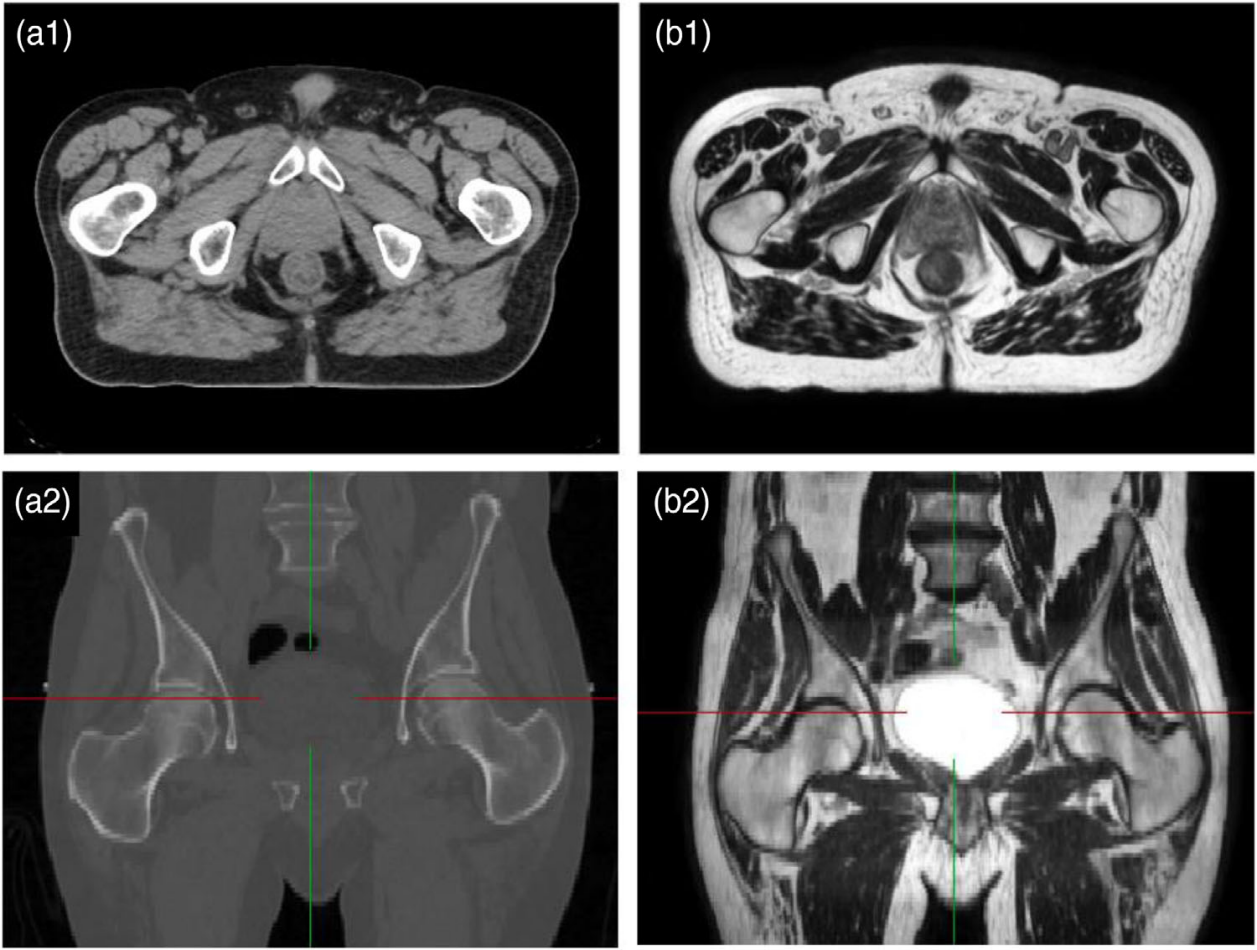
A corresponding pair of axial and sagital CT images shown in Panels A1 and A2 and the corresponding calculated synthetic MRI images shown in panels B1 and B2 for pelvic case.

A pair of axial and sagital CT images (panels A1 and A2) and the corresponding calculated synthetic MRI images (panels B1 and B2) for a pelvic case are shown in Figure 3. Another reason for calculating synthetic MRI images is to help the clinicians with soft tissue definition.^23^, ^24^ This aspect is obvious in the images shown in Figure 3. Image synthesis also allows for a single imaging modality, for example, MRI to provide all the information that was classically obtained from CT and MRI scans and vice versa.

## 4 THE IMAGING WORKFLOW FOR UPRIGHT RADIATION THERAPY TREATMENTS

As stated earlier, there is a lack of high‐magnet‐strength MRI scanners that can scan the patient in the upright orientation. The CT images acquired from an upright CT scanner will provide the density and geometrical information required for the treatment planning, patient positioning and daily beam delivery stages of the treatment. However in many cases the target might not be identifiable on the CT images but will only be visible in the MRI images acquired using a commercially available recumbent MRI scanner (often a high magnetic field strength scanner). Calculating synthetic images provides a solution to this problem following one of two possible approaches, that is, (Method A) calculating synthetic upright MRI images from the upright CT scans or (Method B) calculating synthetic supine CT images from the supine diagnostic MRI images. Both methods are described below.

**FIGURE 4:**
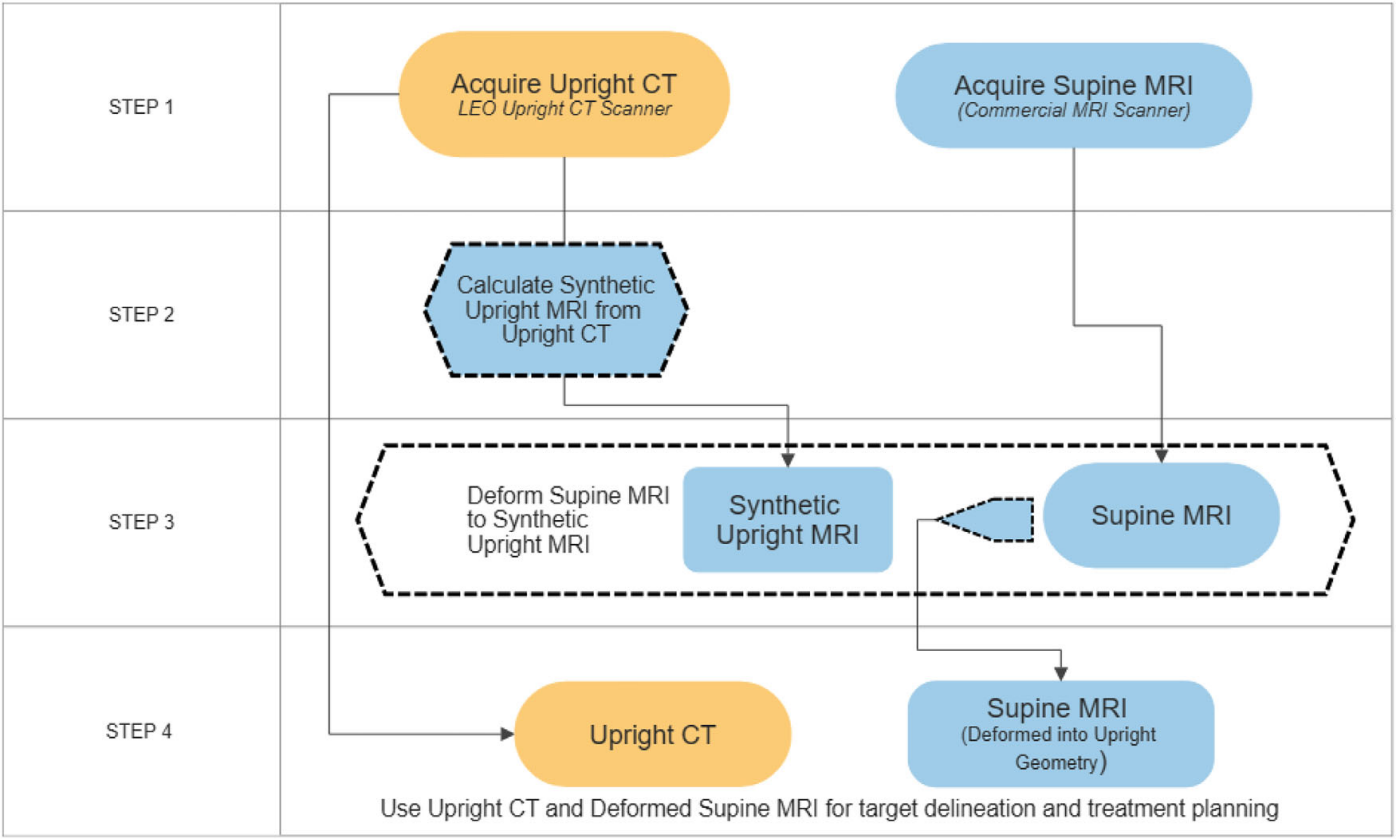
The first imaging workflow (Method A) for upright treatments where a high magnetic field is required for target definition and delineation.

Method A: Calculating synthetic MRI images from the acquired upright CT images. The imaging workflow is illustrated in Figure 4. The imaging workflow starts with acquiring a supine MRI scan and an upright CT scan of the patient inclined to the position that the patient will be treated as is illustrated in Step 1 (first row) of the flow chart shown in Figure 4.

The next step in the workflow (Step 2, Figure 4) is to calculate the synthetic MRI images from the upright CT images. The resultant synthetic MRI images will be in the same geometric space as the upright CT and all the organs will also be in the upright orientation and have the correct shapes and positions. The third step in the workflow comprises of deforming the supine MRI images into the upright geometry using intra‐modality deformations, which are stronger and deliver better results than inter‐modality deformations. As stated above, the latter would have been required if the synthetic MRI images were not available. This results in an upright MRI data set containing the target information, that is, the target info is obtained from the deformed supine MRI images. The final step (Step 4) comprises of transferring the deformed MRI data set together with the upright CT data to the treatment planning system for target delineation and treatment planning in the upright orientation.

The problem with Method A is that the user needs to know the exact imaging sequences that were used to acquire the corresponding diagnostic MRI images in order to calculate the synthetic MRI images since any types of MRI images can be derived from a CT data set, for example, T1 or T2‐weighted images and many more combinations.

**FIGURE 5:**
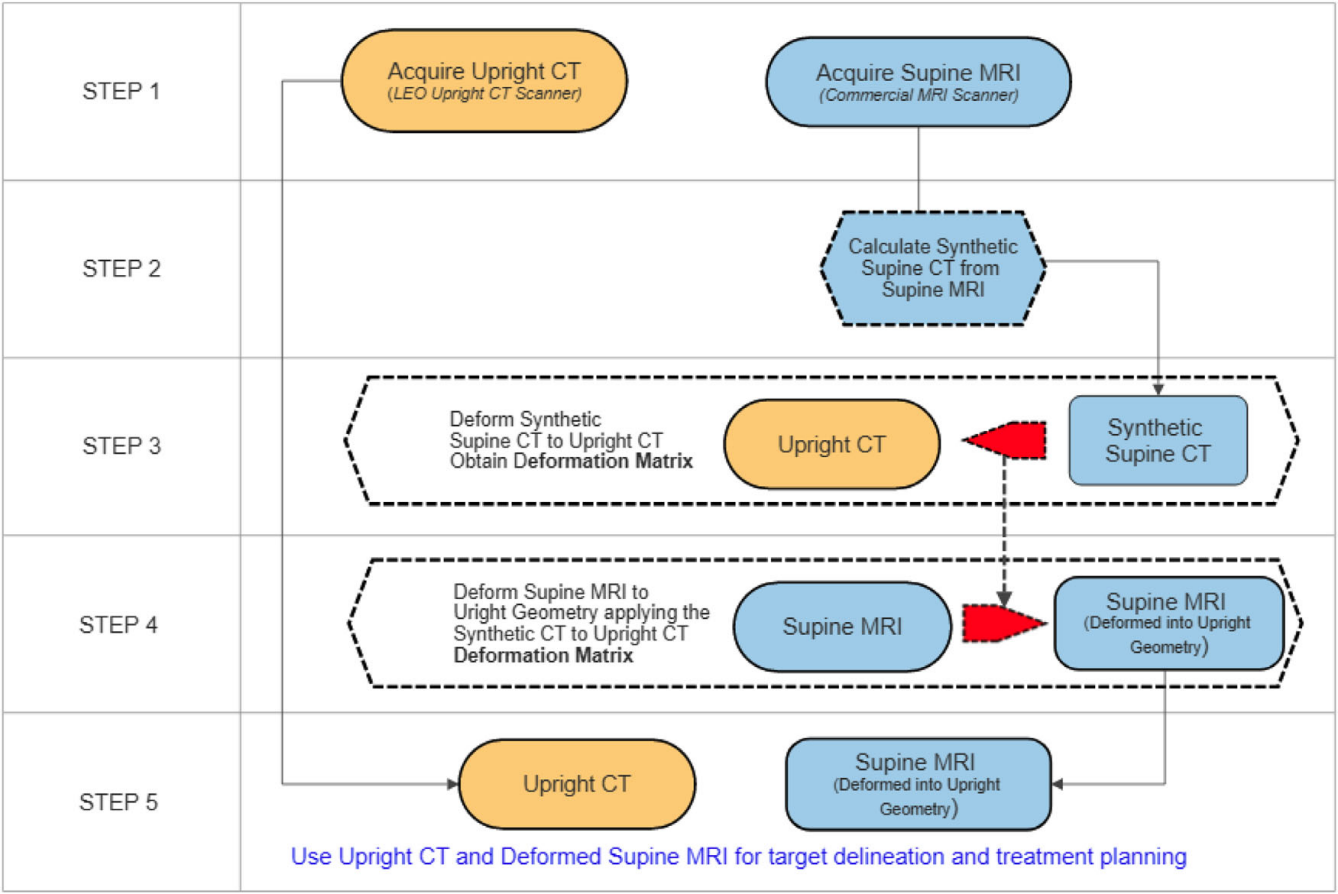
The second imaging workflow (Method B) for upright treatments where a high magnetic field MRI is required for target definition and delineation.

Method B: Calculating synthetic CT images from the acquired supine MRI images. The imaging workflow is illustrated in Figure 5. Similar to method A, the imaging workflow starts with acquiring a supine MRI scan and an upright CT scan of the patient, inclined to the position that the patient will be treated, as is illustrated in Step 1 (first row) of the flow chart shown in Figure 5. The next step in the workflow (Step 2, Figure 5) is to calculate the synthetic CT images from the supine MRI images. In the third step (Step 3, Figure 5) the synthetic supine CT images are deforemed to the upright CT images using intra modality deformations, obtaining the deformation matrix. In the fourth step (Step 4, Figure 5) the deformation matrix obtained in Step 3, is applied to the Supine MRI data set, deforming the supine MRI data into the upright geometry. The resultant deformed MRI images will be in the same geometric space as the upright CT and all the organs will also be in the upright orientation and have the correct shapes and positions. The final step (Step 5) comprises of transferring the deformed MRI data set together with the upright CT data to the treatment planning system for target delineation and treatment planning in the upright orientation.

The main advantage of method B is that only one unique set of CT images can be derived from the acquired MRI images. The only prior knowledge that is typically required by these Synthetic CT models, although it might differ between software solutions, are the MR scan weighting, that is, T1 or T2, that was used to obtain the reference MRI. By definition, CT images are uniquely defined by the tissue densities meaning that only one solution is possible. Note that method B requires an extra step as compared to method A.

## 5 DISCUSSION AND SUMMARY

The workflows described above may solve the lack of high magnetic field MRI imaging in the upright position. The intra‐modality deformable registrations will still require special attention since large changes in organ shapes between the supine and upright orientations, as is illustrated in Figure 2 for the bladder, might still cause some smaller structures, like the prostate, to get deformed incorrectly. However, we believe that the intra‐modality deformation into the upright synthetic MRI will be much easier and more reliable. Cropping the supine MRI images to only contain the volume of interest, for example, the prostate, seminal vesicles, rectum, and a small part of the bladder, reduce the impact of the larger bladder on the deformation map. Either one of the methods can be used but it worth re‐iterating that the second method (Method B) does not require any knowledge of the reference MRI scan sequences (other than the MR scan weighting) since only one unique solution for the CT data set is possible. It does however involve one more computational step, that is, five steps versus only four steps in method A.

## AUTHOR CONTRIBUTIONS

Andries Niek Schreuder: Conceptualization of the concepts described, wrote the paper.

Nikos Paragios: Conceptualization of the concepts described, contributed to writing the paper, provided the synthetic images, and reviewed and edited the paper.

Michael Kissick, Michelle Lis, Tracy S. A. Underwood: Reviewed and edited the paper.

Rockwell Mackie: Conceptualization of the concepts described and critical reviewing and editing of the paper.

## CONFLICT OF INTEREST STATEMENT

Andries Niek Schreuder, Michael Kissick, Michelle Lis, Tracy S. A. Underwood, and Rockwell Mackie have no conflict of interest, but all are full‐time employees of Leo Cancer Care.

Nikos Paragios has no conflict of interest and is the Founder and CEO of Therapanacea.
